# Covalent Organic Framework (COF‐1) under High Pressure

**DOI:** 10.1002/anie.201907689

**Published:** 2019-12-02

**Authors:** Jinhua Sun, Artem Iakunkov, Igor A. Baburin, Boby Joseph, Vincenzo Palermo, Alexandr V. Talyzin

**Affiliations:** ^1^ Department of Physics Umeå University 90187 Umeå Sweden; ^2^ Department of Industrial and Materials Science Chalmers Tekniska Högskola 41296 Göteborg Sweden; ^3^ Theoretische Chemie Technische Universitat Dresden Bergstraße 66b 01062 Dresden Germany; ^4^ Gd R IISc-ICTP Elettra-Sincrotrone Trieste 34149 Basovizza Trieste Italy

**Keywords:** 2D materials, DFT calculations, high pressure, Raman spectroscopy, XRD

## Abstract

COF‐1 has a structure with rigid 2D layers composed of benzene and B_3_O_3_ rings and weak van der Waals bonding between the layers. The as‐synthesized COF‐1 structure contains pores occupied by solvent molecules. A high surface area empty‐pore structure is obtained after vacuum annealing. High‐pressure XRD and Raman experiments with mesitylene‐filled (COF‐1‐M) and empty‐pore COF‐1 demonstrate partial amorphization and collapse of the framework structure above 12–15 GPa. The ambient pressure structure of COF‐1‐M can be reversibly recovered after compression up to 10–15 GPa. Remarkable stability of highly porous COF‐1 structure at pressures at least up to 10 GPa is found even for the empty‐pore structure. The bulk modulus of the COF‐1 structure (11.2(5) GPa) and linear incompressibilities (*k*_[100]_=111(5) GPa, *k*_[001]_=15.0(5) GPa) were evaluated from the analysis of XRD data and cross‐checked against first‐principles calculations.

## Graphical abstract

**Under pressure we're breaking**: High‐pressure synchrotron‐radiation XRD and Raman experiments with mesitylene‐filled and empty‐pore COF‐1 demonstrate the stability of the structure up to 12–15 GPa. Partial amorphization and a collapse of the framework is observed at higher pressure. Experimental bulk modulus and linear incompressibilities of COF‐1 are useful for benchmarking theoretical descriptions of van der Waals interactions in solids.

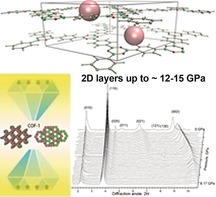

Covalent organic frameworks (COFs) are porous polymers composed only of light elements.^1^ The absence of metal atoms in their structure makes COFs distinctly different compared to metal–organic framework materials (MOFs). COFs are typically synthesized by polycondensation reactions using a variety of organic building blocks providing hundreds of structures with a broad range of pore sizes and surface areas.^1^, ^2^ The most common and historically first discovered COF‐1 is prepared using 1,4‐benzenediboronic acid (DBA) as a precursor molecule.^1^ The structure of COF‐1 consists of benzene rings linked by B_3_O_3_ into hexagon‐shaped 2D sheets, which are stacked into a layered structure, resembling in this respect the structure of graphite composed of graphene layers. By analogy with graphene, the single layer of COF material could be named as COFene since it represents a true 2D material composed of carbon, hydrogen, boron, and oxygen. The structure of COFs with precisely defined pore sizes (e.g., 1.5 nm for COF‐1) is a unique property, which could be useful for applications that require a high surface area and porosity. Therefore, various COFs and COF‐based composites have been proposed for a variety of applications,^3^ for example, in catalysis,^4^ as membrane materials,^5^ and as materials for energy‐related devices.^6^ Most recently, much interest was focused on using single COF layers in combination with other 2D materials, for example, graphene.^7^

However, little is known about mechanical properties of COFs or single layered COFenes except for a few theoretical estimates.^8^ It is interesting to note that the in‐plane compressibility of graphene and bulk modulus of graphite were evaluated using high‐pressure methods^9^ long before the explosion of interest in isolated graphene in the early 2000s. High‐pressure studies of several other materials composed of rigid 2D layers held together by weak van der Waals bonding have been reported over past 15 years, including for example, turbostratic boron nitride, graphite oxides,^10^ and Mxenes.^11^ Surprisingly, no high‐pressure studies are so far available for layered COFs. That is despite rather strong interest in the pressure‐dependent properties of various porous MOF structures. A variety of new phenomena have been reported for MOFs at high pressures, including for example, pressure‐driven collapse of structure,^12^ phase transitions,^13^ anomalies related to pore filling with guest molecules,^14^ negative linear compressibility,^15^ and others.15b Very recently, exploration of MOFs was extended also to experiments combining high pressure with high temperatures.^16^ However, most of the MOFs studied at high pressure show rigid covalent 3D framework structure unlike the layered COF‐1.

The COF‐1 structure is formed by staggered AB‐arrangement of layers, analogous to the packing of graphene layers in graphite (Figure 1). According to the standard synthesis procedure, the pores of COF‐1 structure are occupied by mesitylene, the solvent involved in solvothermal synthesis.^17^ Vacuum annealing of COF‐1 allows removal of the guest molecules, providing the structure with interconnected pore network and high specific surface area (ca. 700 m^2^ g^−1^ by N_2_ BET). It is believed that the COF‐1 structure is re‐arranged from staggered into eclipsed form as a result of solvent evaporation. The eclipsed stacking of COF‐1 layers is formed when atoms of adjacent sheets are located over each other, possibly with a slight relative shift.^1^, ^17^, ^18^ However, strong disorder is typical for the annealed COF‐1 structure, providing only few asymmetric XRD reflections and preventing precise structure analysis.^1^, ^18^

**Figure 1:**
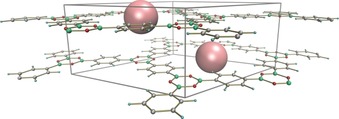
Crystal structure model of COF‐1‐M with pores filled by disordered mesitylene molecules (balls).

Herein, we present the first (to our knowledge) high‐pressure study of the empty pore COF‐1 and mesitylene pore‐filled (COF‐1‐M) materials. Both materials reveal an irreversible transformation into a semi‐amorphous high‐pressure phase around 12–15 GPa, similar to the phase transformation observed in graphite. However, the pristine COF‐1 phase can be recovered even after compression up to 10–12 GPa thus providing an example of a remarkable pressure stability for highly porous structure. Test experiments with high‐temperature treatment at 7.7 GPa demonstrate decomposition of COF‐1 structure.

COF‐1‐M material was synthesized in two batches, both showing good agreement with earlier published data as confirmed by XRD, XPS, Raman and FTIR spectra and TGA (see the Supporting Information). Indexing of XRD patterns using the *P*6_3_/*mmc* space group provides *a*=15.12 Å and *c*=6.627 Å for the first batch and *a*=15.16 Å and *c*=6.614 Å for the second batch. High‐pressure experiments performed in DAC showed that the ambient pressure phase is stable at least up to circa 13–15 GPa (Figure 2).

**Figure 2:**
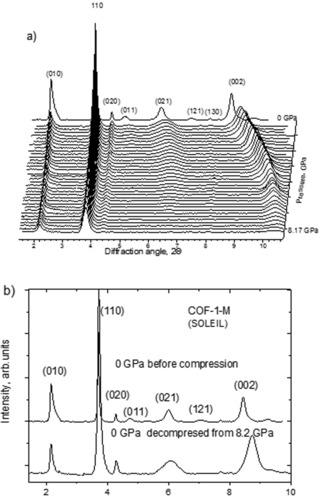
XRD patterns recorded using COF‐1‐M in DAC (no pressure medium). a) Compression up to 8.2 GPa (*λ*=0.4859 Å) b) XRD patterns recorded before compression and after decompression (background subtracted).

Compression of COF‐1‐M structure results in a relatively small decrease of the *a*‐parameter (down to 14.4 Å at 15 GPa) reflecting the rigid covalently bound structure of COFene layers and much stronger change in the *c*‐parameter reflecting the weaker van der Waals bonding between the layers. The distance between COFene layers decreases from 3.31 Å down to 2.65 Å at 15 GPa (Figure 3). Compression of COF‐1‐M results in a strong intensity decrease and broadening of (002) reflection already starting from relatively small pressures. Moreover, other reflections which include ℓ‐indexes could not be followed above 5–7 GPa. Adding mesitylene as a pressure medium has not provided significant differences in pressure dependence of the unit cell parameters of COF‐1‐M, Figure 3. The data recorded with small pressure steps up to 7 GPa were used to evaluate the bulk modulus of COF‐1‐M structure and compressibility of COFene layers.

**Figure 3:**
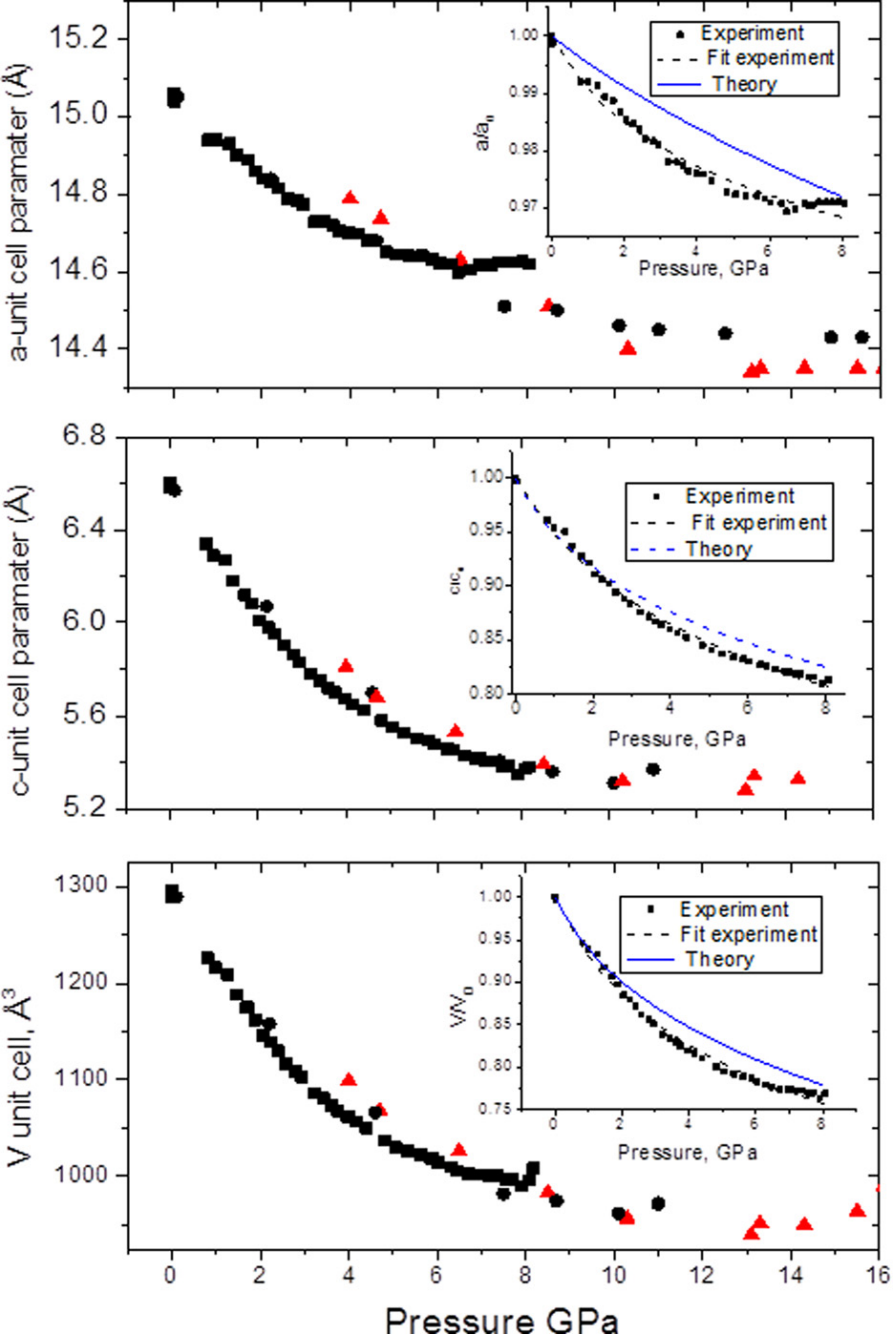
Pressure dependences of unit cell parameters and unit cell volume of COF‐1‐M recorded in three separate experiments: ▪ and • without pressure medium, ▴ in mesitylene. Insets show experimental compressibility of graphite oxide in the *a*‐ and *c*‐ directions with fitting by Murnaghan equation and theoretical data.

The bulk modulus and its pressure derivative (*B*=11.2(5) GPa and *B*′=6.0(3)) were estimated from a least‐squares fit to the Murnaghan equation of state. Linear incompressibilities, as defined by Equations (1), (2):

(1) $$k_{\left[100\right]}=-{\left(\frac{dp}{d\ln a}\right)}_{p=0}=111\mathrm{GPa}$$

(2) $$k_{\left[001\right]}=-{\left(\frac{dp}{d\ln c}\right)}_{p=0}=15\mathrm{GPa}$$

were found from a fit to a one‐dimensional analogue of the Murnaghan equation. From the Equations (3), (4):

(3) $$B=\frac{C_{33}\left(C_{11}+C_{12}\right)-2C_{13}^{2}}{C_{11}+C_{12}+2C_{33}-4C_{13}}$$

(4) $$\frac{k_{\left[100\right]}}{k_{\left[001\right]}}=\frac{\left(C_{11}+C_{12}\right)-2C_{13}}{C_{33}-C_{13}}$$

and assuming *C*_13_≈0, we can estimate *C*_11_ + *C*_12_≈105 GPa and *C*_33_≈14 GPa.^19^ We notice a good agreement between experiment and dispersion‐corrected density‐functional theory calculations (at the PBE‐D3(BJ) level) with respect to bulk modulus and the *C*_33_ constant (theory: *B*=11.4 GPa and *C*_33_=13.8 GPa, see the Supporting Information) but a theoretical value of the in‐plane elastic modulus is significantly higher (*C*_11_ + *C*_12_=156 GPa), pointing towards the existence of in‐plane defects.

Comparing elastic properties of graphite and COF‐1, we notice a drastic drop in the in‐plane stiffness, as evidenced by difference in *k*_[100]_ (111(5) GPa and 1250(70) GPa9b for COF‐1 and graphite, respectively). Therefore, COFene layer is about 10‐fold softer than graphene. However, the difference in the bulk moduli of COF‐1 and graphite corresponds approximately to the reduction in the densities (ρ_graphite_/ ρ_COF‐1‐M_≈1.8; *B*_graphite_/*B*_COF‐1‐M_≈3.3). A similarly strong difference in mechanical properties can also be seen between COF‐1‐M and h‐BN, although the latter is a bit softer than graphite (C_11_+C_12_=980 GPa, bulk modulus 25.6 GPa).^20^ In fact, the bulk modulus of COF‐1‐M resembles that of a molecular crystal or a MOF with a similar density,^21^ indicating that overall compressibility is dominated by van der Waals interactions. Note that COF‐1 sheet edges are likely to be terminated by B‐OH groups which makes it rather different compared to, for example, graphite or h‐BN.

Phase transformation occurs in COF‐1‐M at around 13–15 GPa as evidenced by visual observation of color change, XRD (Figure 4), and Raman spectroscopy data. The ambient pressure COF‐1 is a white transparent powder. As a result of the phase transformation, the color changes to deep brown and the material become less transparent (see image in the Supporting Information).

**Figure 4:**
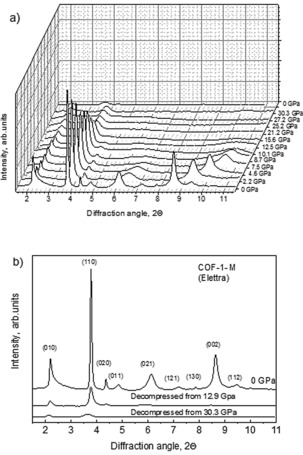
a) XRD patterns recorded from COF‐1‐M upon compression up to 30.3 GPa (*λ*=0.4957 Å) and b) XRD patterns recorded before compression and after decompression.

Partial amorphization of the material is evident in XRD patterns recorded above the transition point with only two weak reflections, (010) and (110), visible in XRD patterns above 15 GPa and preserving up to 30.3 GPa (Figure 4).

As shown in Figure 2 decompression from 8.2 GPa results in a complete recovery of the ambient pressure phase (with slightly different unit cell parameters), decompression from 12.9 GPa is only partly reversible, while compression up to 30.3 GPa is clearly not reversible, Figure 4 b. The high‐pressure phase is preserved after release of pressure, exhibiting only two weak XRD reflections, which roughly correspond to (010) and (110) of the ambient phase.

Similar results were also observed for COF‐1 material annealed to remove guest solvent molecules. The empty pore COF‐1 shows strong but asymmetric (010) reflection at ambient pressure indicating the in‐plane disorder induced by evaporation of guest molecules. The intensity of the (010) reflection decreases very strongly already upon rather mild compression, whereas the asymmetric part decreases in intensity less strongly thus providing two independent components, Figure 5. The dataset recorded for empty‐pore COF‐1 is less suitable for the evaluation of unit cell parameters, bulk modulus, and linear compressibility due to the rather broad and asymmetric shape of XRD reflections. Nevertheless, the data collected in high‐pressure experiments with the empty pore COF‐1 are very similar to those of the pore filled COF‐1‐M (see the Supporting Information).

**Figure 5:**
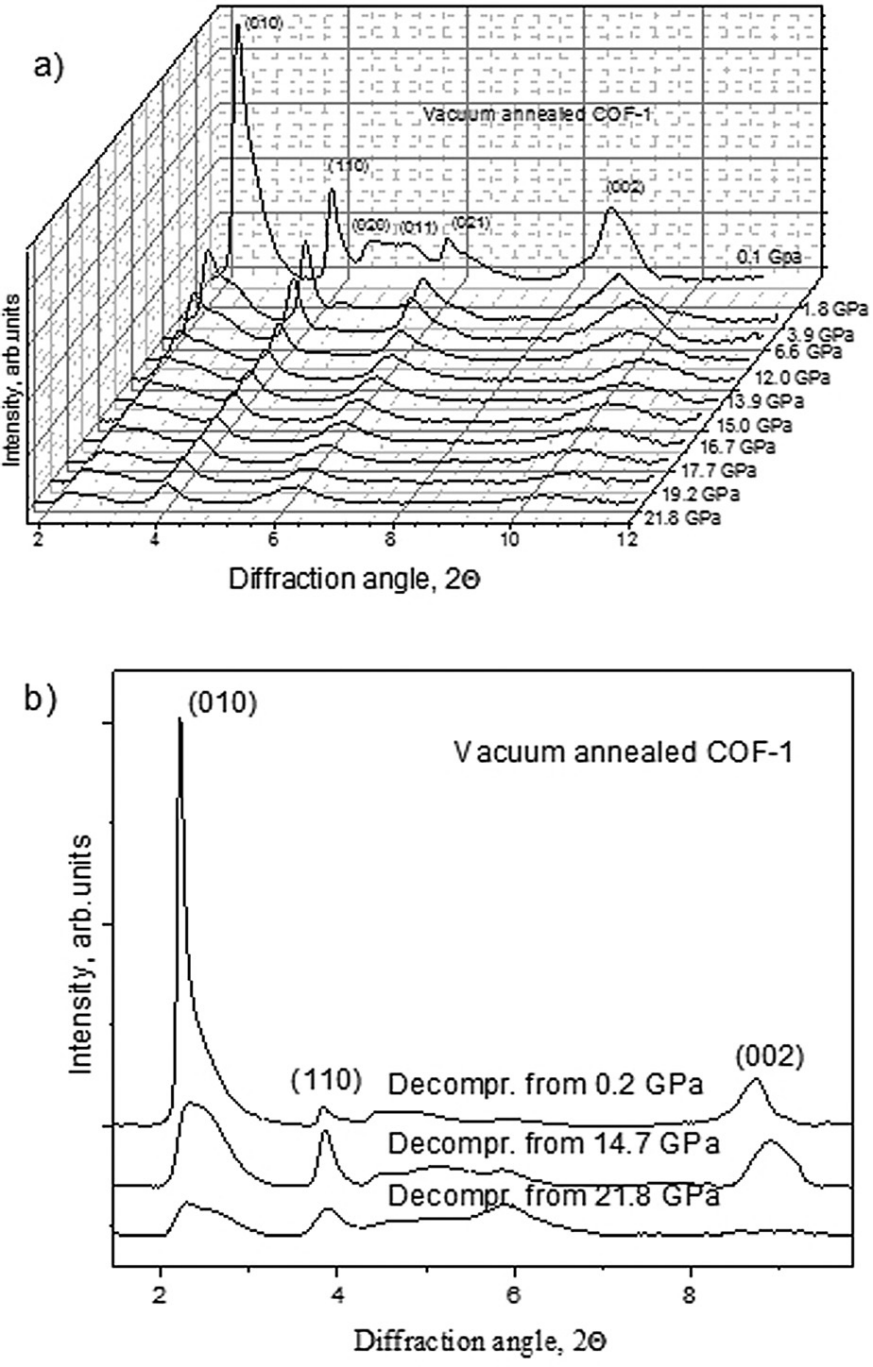
XRD patterns recorded upon compression of annealed empty‐pore COF‐1 up to 21.8 GPa and XRD patterns recorded in open cell after decompression in three separate experiments (*λ*=0.4957 Å).

Strong compressibility is observed along the c‐direction down to *c* values of 5.4 Å at 12–15 GPa. The *a*‐unit cell parameter was found to decrease somewhat more strongly compared to the material with filled pores with minimal values observed in two separate experiments 13.7 Å and 13.9 Å at 12 GPa. Adding mesitylene to the empty pore COF‐1 has not resulted in a significant change of compressibility (see the Supporting Information).

The ambient pressure empty‐pore COF‐1 phase was recovered after compression up to pressures below circa 15 GPa, while irreversible transformation occurs at higher pressures. The sample decompressed from 21.8 GPa showed no (002) reflection, thus demonstrating the absence of ordering in the *c*‐direction.

Annealed COF‐1 is a highly porous material with relatively large BET surface area, but it shows remarkable stability at high‐pressure conditions. The material is partly recovered even after compression up to 14.7 GPa, a pressure that significantly exceeds the collapse point for most other porous materials. For example, rather rigid carbon nanotubes were demonstrated to deform already at 1.5–1.7 GPa^22^ with a complete pore collapse at 2–13 GPa^23^ depending on their diameter.^24^

However, Raman spectra recorded of the empty pore COF‐1 upon compression to 10.2 GPa and after decompression show that the ambient pressure structure is preserved (Figure 6).

**Figure 6:**
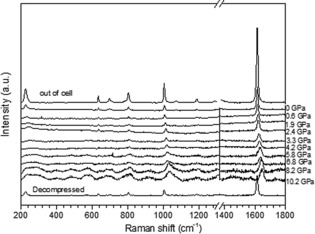
Raman spectra recorded from COF‐1 (annealed) upon compression to 10.2 GPa and after decompression using 514 nm laser (background subtracted).

The extreme pressure stability of the empty pore COF‐1 is related to a high (for a porous solid) in‐plane Young's modulus (*E_a_*≈*C*_11_−*C*_12_^2^/*C*_11_≈33 GPa). However, low shear moduli parallel to the basal planes *C*_44_<1 GPa, as well as for individual layers (*C*_11_−*C*_12_)/2≈9 GPa (Supporting Information) are responsible for loss of ordering between layers at relatively low pressures of a few gigapascals.

The nature of the high‐pressure phase formed from COF‐1 (both empty pore and filled pores structures) above 15 GPa cannot be unambiguously resolved using our results owing to the partial amorphization of the material. Interpreting only XRD data, one could possibly suggest a structure composed of deformed COFene layers with a complete stacking disorder. However, Raman spectroscopy shows that the ambient pressure COF‐1 layer structure is not preserved above 15 GPa (Figure 7).

**Figure 7:**
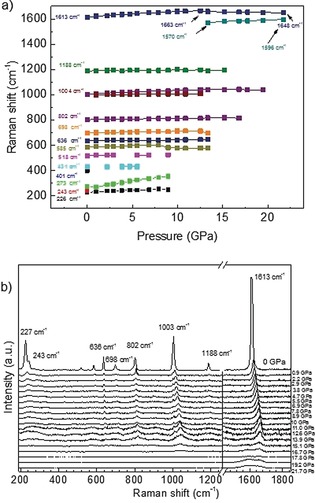
Raman spectra (514 nm laser excitation) recorded from COF‐1‐M upon compression to 21.7 GPa (background subtracted).

The pressure dependence of Raman peaks originating from ambient COF‐1 can be followed up to circa 15 GPa. Dramatic changes are observed in the Raman spectra at higher pressures, most of the peaks disappear and a new feature appears at 1570 cm^−1^ starting from 13.3 GPa. Only two broad features are observed at 21.7 GPa, 1648 cm^−1^ and 1596 cm^−1^. The point of transition is also marked by the change of slope for the pressure dependence of the in‐plane C−C peak position, it is found at 1613 cm^−1^ at ambient pressure, upshifts to maximal value of 1663 cm^−1^ at 12.5 GPa and downshifts at higher pressures. Unfortunately, Raman spectra of the high‐pressure phase could not be recorded after decompression owing to very strong luminescence background observed with the two available lasers (514 nm and 633 nm).

The most likely reason for the changes in Raman spectra and XRD patterns observed in the 12–15 GPa region is the collapse of the COF‐1 structure. In fact, the Raman spectra recorded at 21.7 GPa are remarkably similar to the Raman spectra recorded from various amorphous carbon nanostructures, for example, carbon onions exhibit two broad peaks at similar positions (17 GPa).^25^ Therefore, we argue that the COF‐1 structure breaks up above 15 GPa.

The collapse of the COF‐1 structure results in the formation of an amorphous phase, which is likely to consist of a complex mixture of B_3_O_3_‐benzene framework fragments, possibly with some fraction of graphitic carbon as indicated by FTIR spectra recorded from decompressed sample (Supporting Information). However, a precise evaluation of collapsed COF‐1 structure requires further experiments. The exact nature of amorphous pressure‐collapsed framework materials is notoriously difficult to reveal and remains unknown for most MOF materials.14a, 26

In conclusion, empty‐pore COF‐1 and mesitylene‐filled COF‐1‐M materials were analyzed at high‐pressure conditions using synchrotron XRD and Raman spectroscopy up to 21.8 GPa and 30 GPa, respectively. Both structures are found to be preserved and reversibly recovered after compression up to circa 12–15 GPa. The pressure dependence of volume (up to 8 GPa) and zero‐stress elastic constants of both empty‐pore and mesitylene‐filled structures of COF‐1 were successfully modelled through first‐principles calculations. Phase transformation into a new phase is detected by formation of brown colored and less‐transparent phase above 13–15 GPa. The high‐pressure phase is semi‐amorphous, it exhibits only two weak XRD reflections, while the Raman spectra provide evidence for the collapse of in‐plane framework structure. Elastic properties of COF‐1 structure and COFene layers were evaluated using experimental data providing the bulk modulus of 11.2(5) GPa and in‐plane linear incompressibility of 111(5) GPa, which are significantly smaller compared to respective values for graphite (*B*=33.8(3) GPa and *k*_[100]_=1250(70) GPa).9b To our knowledge these are the first experimental data on the mechanical properties of layered COF systems, which could be used to benchmark recently developed theoretical approaches to account for van der Waals interactions in solids.^27^

## Conflict of interest

The authors declare no conflict of interest.

## Supporting information

As a service to our authors and readers, this journal provides supporting information supplied by the authors. Such materials are peer reviewed and may be re‐organized for online delivery, but are not copy‐edited or typeset. Technical support issues arising from supporting information (other than missing files) should be addressed to the authors.

Supplementary
