# Supplementary material for: Covalent Organic Framework (COF‐1) under High Pressure
Source: Angew Chem Int Ed Engl. 2019 Dec 2;59(3):1087–92. doi: 10.1002/anie.201907689 (PMC7065212; doi:10.1002/anie.201907689)
Supplement: Supplementary file 1 — Supplementary [file ANIE-59-1087-s001.pdf]

Angewandte Chemie
Eine Zeitschrift der Gesellschaft Deutscher Chemiker

Supporting Information

Covalent Organic Framework (COF-1) under High Pressure

*Jinhua Sun, Artem Iakunkov, Igor A. Baburin, Boby Joseph, Vincenzo Palermo, and Alexandr V. Talyzin**

anie_201907689_sm_miscellaneous_information.pdf

Supporting information

1. Experimental section.

2. DFT calculations.

3. Characterization of COF-1 materials used for high pressure experiments: XRD, Raman spectra, TGA, Nitrogen sorption analysis (surface area), FTIR, XPS, SEM.

4. XRD data recorded in DAC for COF-1 and COF-1-M compressed in mesitylene.

5. Compressibility data for empty pore (annealed) COF-1.

6. Additional data for analysis of Raman spectra.

7. FTIR analysis of decompressed samples.

8. XPS analysis of sample decompressed from 23.2 GPa.

1. Experimental Section

Synthesis of COF-1 was performed according to Ref.1. Characterization by XRD, Raman spectroscopy, FTIR, XPS and TGA confirms successful synthesis of materials. SEM images showed that the powder is composed by small platelet shaped crystal with typical size ~50-200 nm and thickness ~10-50 nm. High pressure experiments were performed in four post or six post DAC cells using stainless steel and rhenium gaskets and culet sizes of diamonds 0.3-05 mm. Powder samples of COF-1 and COF-1 were studied without pressure medium since e.g. gases could act as guest species in the porous structure. Mesitylene was added in some experiments as the solvent used in synthesis of material. First set of experiments was recorded using synchrotron radiation at PSICHE beamline (SOLEIL), the wavelength of λ = 0.4859Å, using a MARE Research image plate detector. The two-dimensional XRD patterns were integrated using Dioptas 3.1 or Fit2D software. Second set of experiments was recorded at Xpress beamline (Elettra) using mar-345 detector and radiation wavelength λ=0.4957Å. Raman spectra were recorded using Renishaw spectrometer with 50* objective. Pressure was calibrated using gold wire (0.005 mm thickness) and ruby (J.C.Chervin Cr^{3-}doped balls) fluorescence. Sample decompressed from 23.3 GPa was also studied *ex situ* using FTIR and XPS (see SI file). DFT calculations were performed with the PWscf/Quantum ESPRESSO package (www.quantum-espresso.org). The PBE functional together with the Grimme dispersion correction and the Becke-Johnson (BJ) damping function PBE-D3(BJ) was chosen since it describes well the elastic properties of layered materials [e.g. hexagonal boron nitride and graphite,[1]. The Kohn–Sham equations were solved using the plane-wave pseudopotential approach (PAW pseudopotentials were taken from the online library https://www.quantum-espresso.org/pseudopotentials). A kinetic energy cutoff of 80 Ry and a density cutoff of 500 Ry were applied to achieve converged results. Variable cell, constant pressure relaxation was performed to optimize the COF-1 structures (both empty pore and mesitylene-filled) in the pressure range 0-8 GPa. In the optimized structures the residual forces were smaller than 0.01 eV/Å and the pressure converged within 0.05 GPa. A standard Monkhorst–Pack *k*-mesh (2 x 2 x 4) was used for the summation over the Brillouin zone. Elastic constants were found from a quadratic fit of energy vs. strain, as explained e.g. in ref. [2]

2. DFT calculations

DFT calculations were performed with the PWscf/Quantum ESPRESSO package (www.quantum-espresso.org). The PBE functional along with the Grimme dispersion correction [D3 with the Becke-Johnson (BJ) damping] was chosen since it describes well the elastic properties of layered materials [e.g. graphite and hexagonal boron nitride[1]]. The Kohn–Sham equations (without spin polarization) were solved using the plane-wave pseudopotential approach (PAW pseudopotentials from the online library https://www.quantum-espresso.org/pseudopotentials were used). A kinetic energy cutoff of 80 Ry and a density cutoff of 500 Ry were applied to achieve converged results. Variable cell, constant pressure relaxation was performed to obtain optimized structures in the pressure range 0-8 GPa. In the optimized structures the residual forces were smaller than 0.01 eV/Å and the pressure converged within 0.05 GPa. A standard Monkhorst–Pack *k*-mesh (2 x 2 x 4) was used for the summation over the Brillouin zone. Elastic constants were found from a quadratinc fit of energy vs. strain, as explained e.g. in Phys. *Rev. B* 51, 17431 (1995).

Table S1. Elastic moduli for COF-1-M (AB polytype)

Elastic moduli, GPa	C_{11}	C_{12}	C_{13}	C_{33}	C_{44}
	90.3	65.8	–1.2	13.8	0.8

Table S2. Elastic moduli for empty pore COF-1 (AA' stacking with slightly displaced layers, Ref. 18)

Elastic moduli, GPa	C_{11}	C_{12}	C_{13}	C_{33}	C_{44}
	89.0	70.5	–0.3	8.7	0.5

3. Characterization of COF-1 materials used for high pressure experiments.

Two batches of COF-1 material were used in our experiments at SOELIL in 2016 and at ELETTRA in 2019. XRD characterization demonstrates only some slight differences between these two batches as shown below.

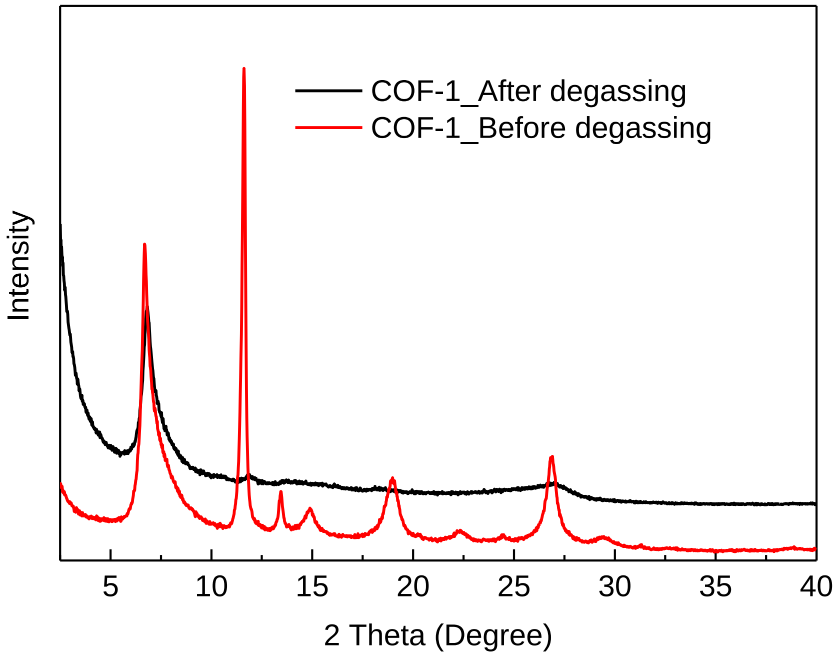

Figure 1S XRDpatterns recorded from COF-1 (2016) before and after vacuum annealing at 250^{0}C. The data are in good agreement with ref. 1

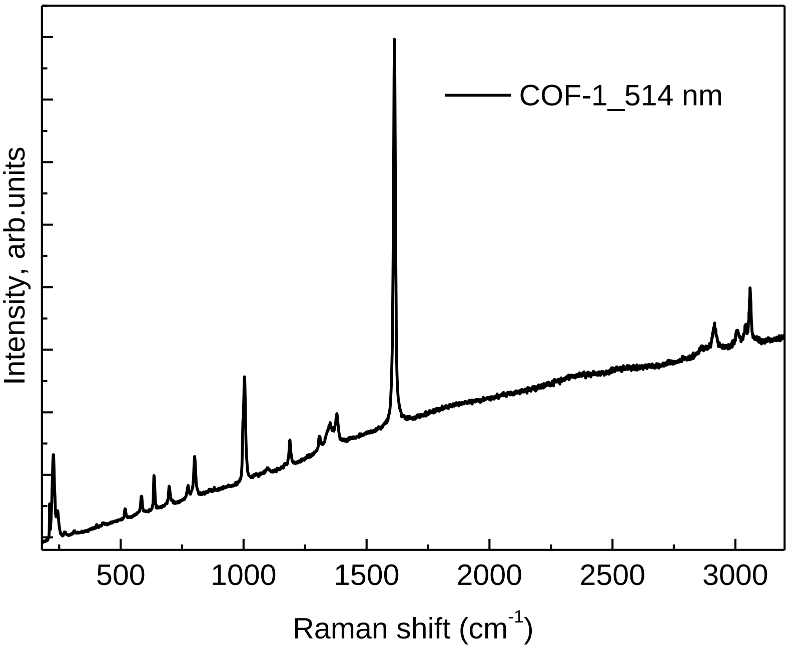

Figure S2 Raman spectrum of COF-1 (batch 2016)

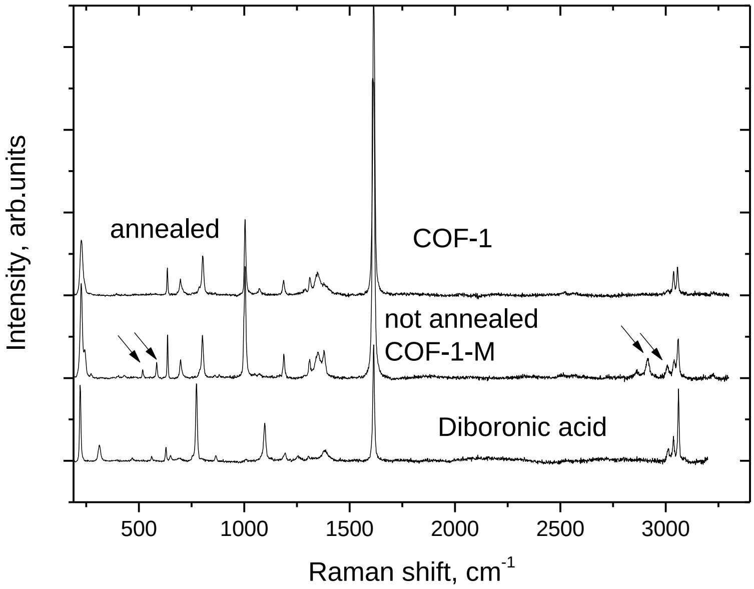

Figure S3. Raman spectra of COF-1 synthesized in 2019 before and after annealing. Spectrum of precursor 1,2-diboronic acid is shown as a reference. Arrows point to the peaks originating from mesitylene.

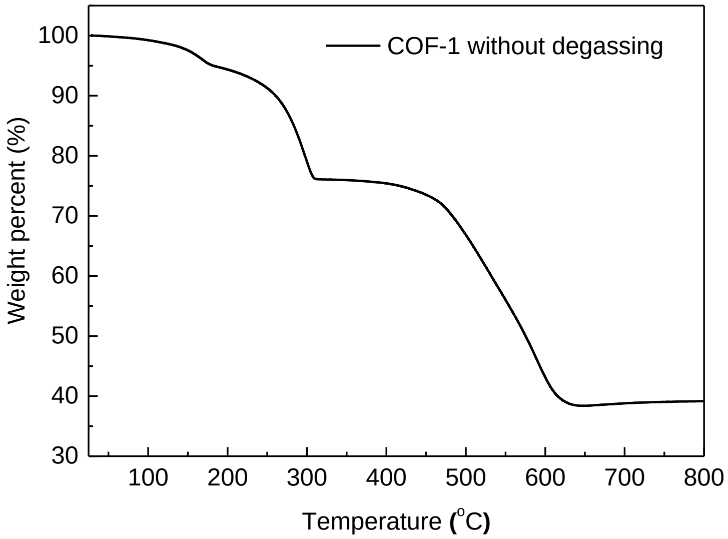

Figure S4 TGA of pristine COF-1 powder recorded in nitrogen flow.

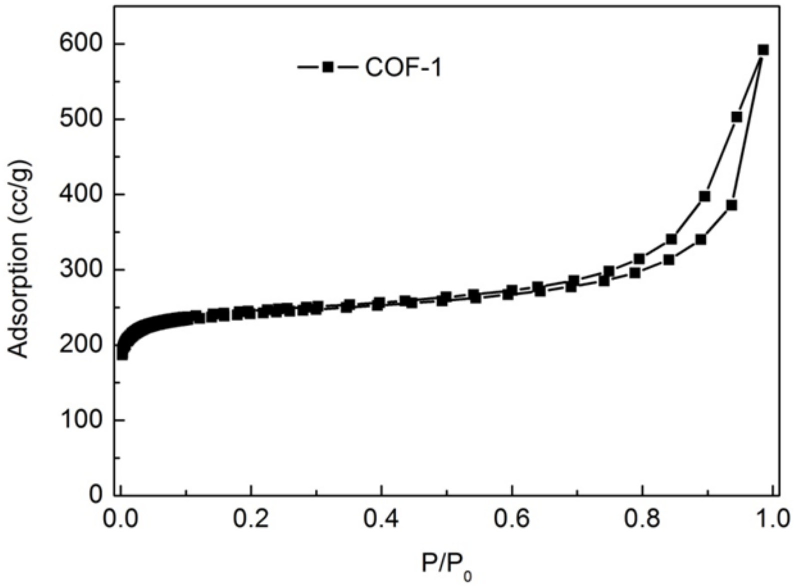

Figure S5. Nitrogen sorption isotherm recorded using annealed COF-1. Analysis of this isotherm provides BET surface area value 964 m^2/g.

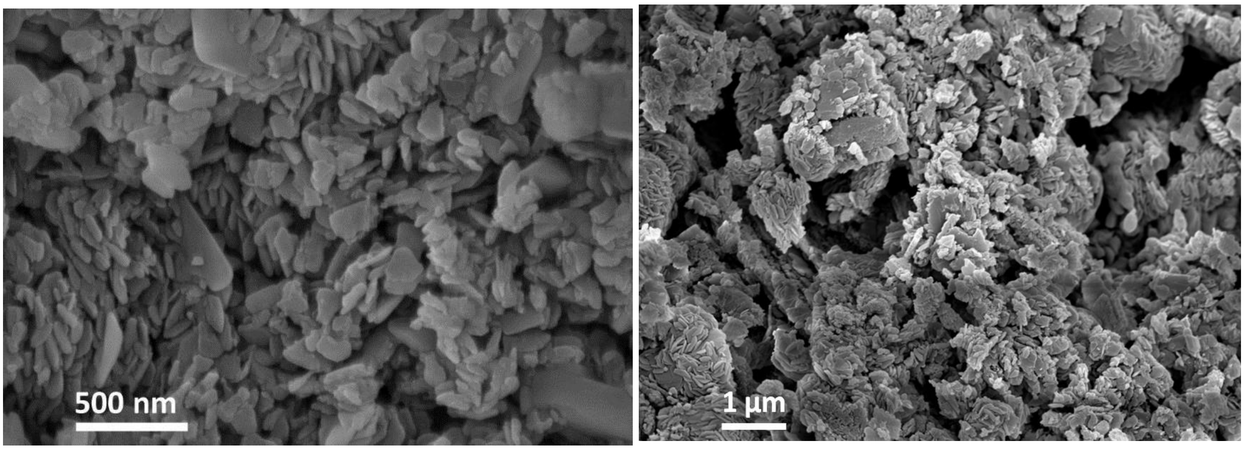

Figure S6 Typical SEM images of lightly grinded COF-1 powder. The crystals are typically platelet shaped with typical diameter of about 50-200 nm and typical thickness of 10-50 nm. Some micrometer sized crystal aggregates are also found. SEM images were recorded using Zeiss Merlin FEG-SEM microscope JEOL JSM-7800F Prime.

a)

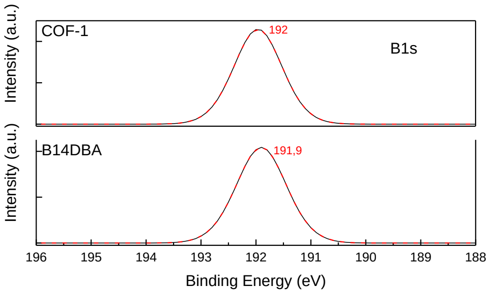

b)

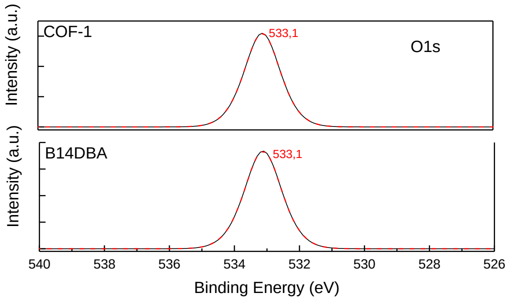

c)

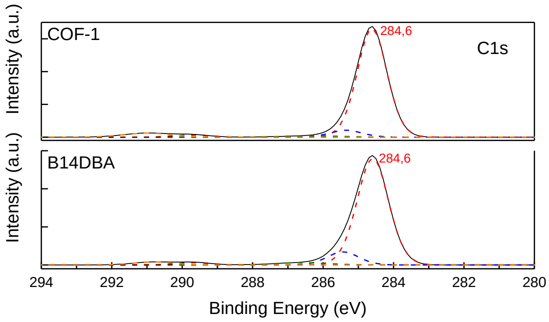

Figure S7. XPS spectra recorded from empty pore COF-1 (a) B 1s, (b) O 1s, and (C) C1s. The sample is from the first batch used in high pressure experiments in 2016. Notably only one peak is observed form oxygen and boron thus providing evidence for purity of the synthesized COF-1. XPS spectra of precursor 1,2-diboronic acid are shown as a reference. Note that boron

is found in COF-1 structure in B_3O_3 units and on the edges the structure is terminated by $B(OH)_2$ as in precursor 1,2-diboronic acid. These two kinds of boron can not be resolved in XPS.

The XPS spectra were recorded with a Kratos Axis Ultra electron spectrometer equipped with a delay line detector. A monochromated Al Kα source operated at 150 W, a hybrid lens system with a magnetic lens, providing an analysis area of 0.3 × 0.7 mm, and a charge neutralizer were used for the measurements. The binding energy scale was adjusted with respect to the C 1s line of aliphatic carbon, set at 285.0 eV. All spectra were processed with the Kratos software.

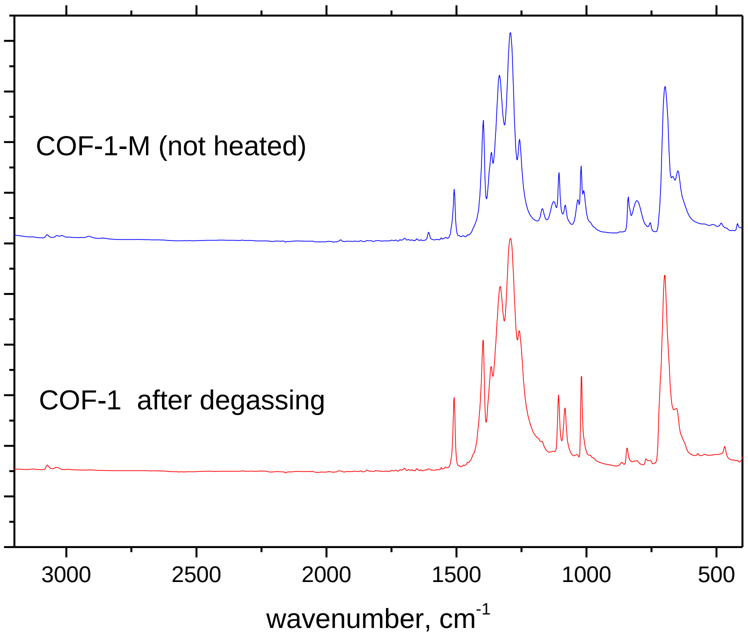

Figure S8. FTIR spectra recorded from precursor COF-1M and COF-1 powders at vacuum conditions using Bruker Vertex 80v FT-IR spectrometer with DTGS detector at vacuum in Attenuated total reflection. The spectra are in excellent agreement with literature data (A.P.Cote et al, *Science* 2005, 310, 1166-1170,[3]).

4. XRD data recorded in DAC for COF-1 and COF-1-M compressed in mesitylene

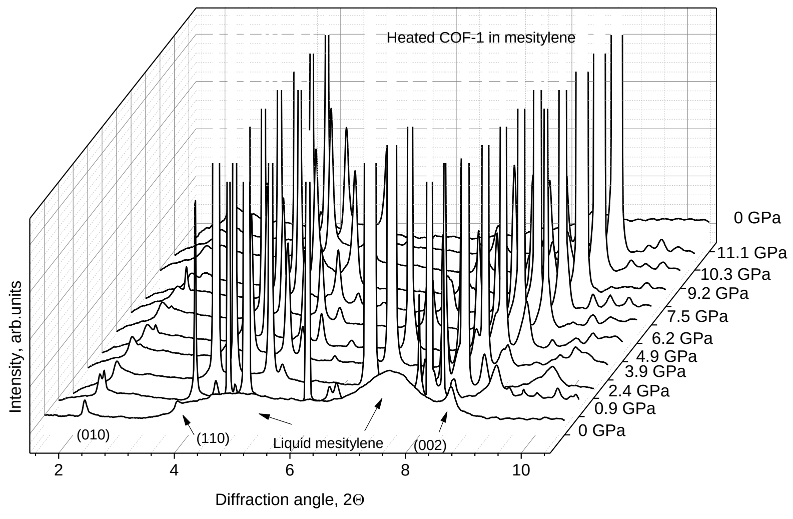

Figure S9. XRD patterns recorded from annealed COF-1 immersed in excess of mesitylene as pressure medium. Multiple strong peaks from solid mesitylene are observed starting from 0.9 GPa.

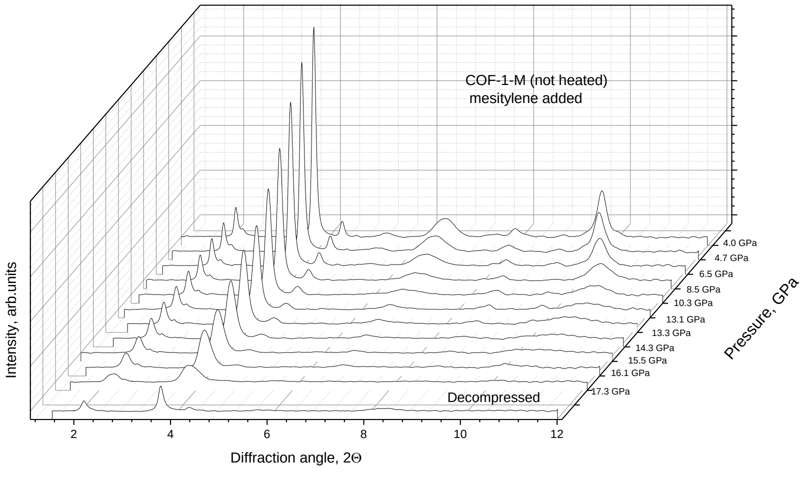

Figure S10. XRD patterns recorded from COF-1-M compressed after addition of mesitylene to the powder pressed into the gasket hole.

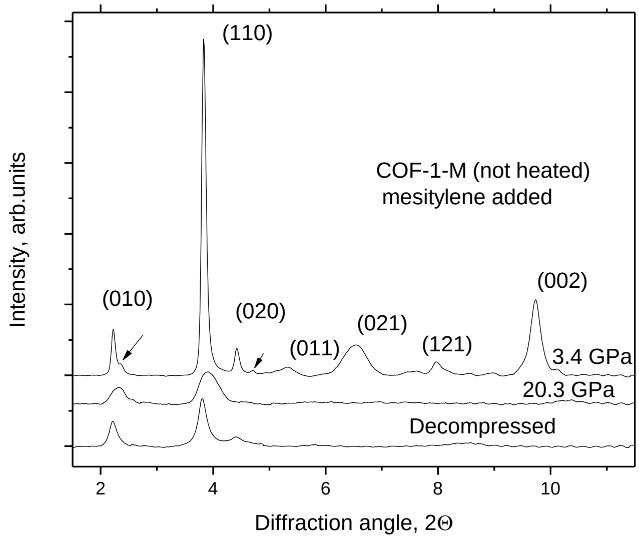

Figure S11. XRD patterns recorded from COF-1-M compressed after addition of mesitylene at 3.4 GPa, maximal pressure of 20.3 GPa and after decompression in closed cell. Irreversible transformation is obvious from the disappearance of all reflections which include ℓ-indexes.

5. Compressibility data for empty pore (annealed) COF-1.

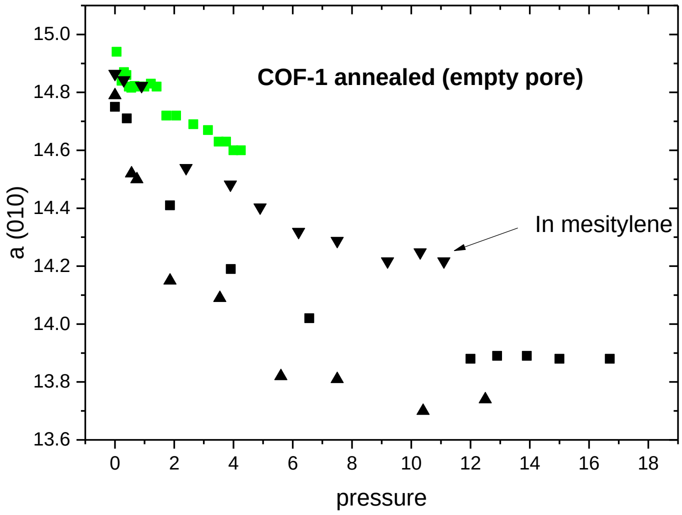

Figure S12. Pressure dependence of a-unit cell parameter for annealed empty pore COF-1. Experiment performed with material synthesized in batch 1 (green) and batch 2 (black symbols). Compression in excess of mesitylene was calculated using position of single (100) reflection.

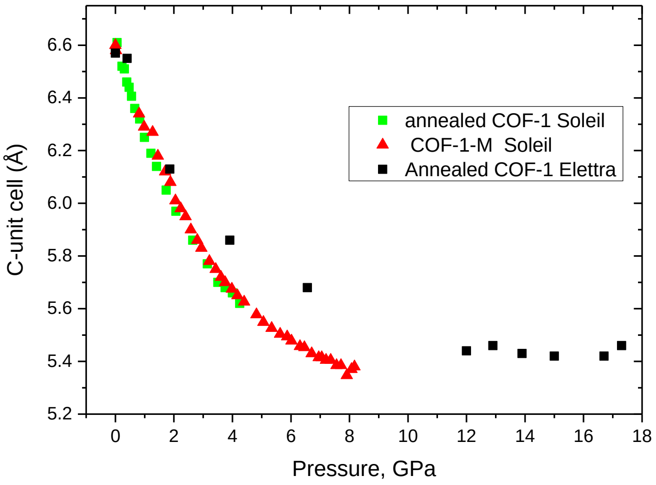

Figure S13 Pressure dependence of c-unit cell parameter for annealed empty pore COF-1. Compressibility of COF-1-M in mesitylene could not be followed in C-direction due to heavy overlap with XRD reflections of solid mesitylene.

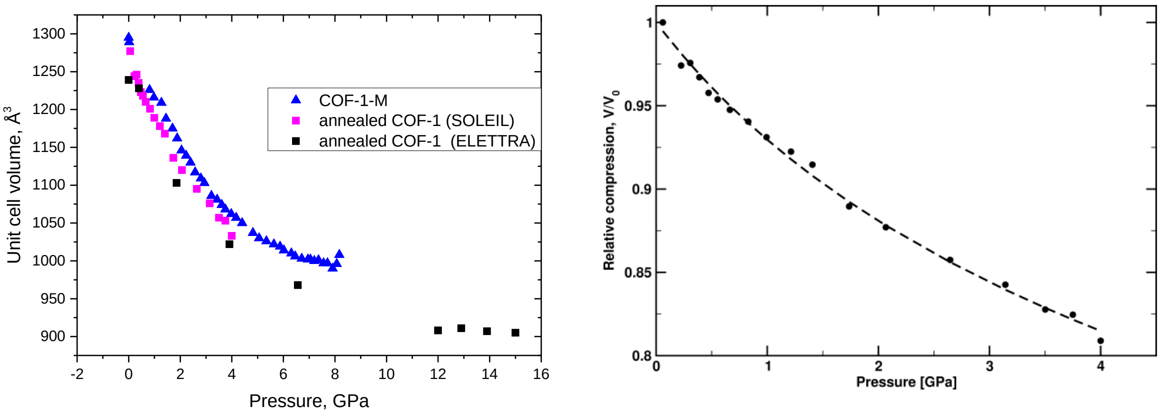

Figure S14 a) Pressure dependence of c-unit cell parameter for annealed empty pore COF-1. Compressibility of COF-1-M in mesitylene could not be followed in C-direction due to heavy overlap with XRD reflections of solid mesitylene. b) Fit of experimental V/V_0 data using Murnaghan equation for the best set of data up to 4 GPa

6. Additional data for analysis of Raman spectra.

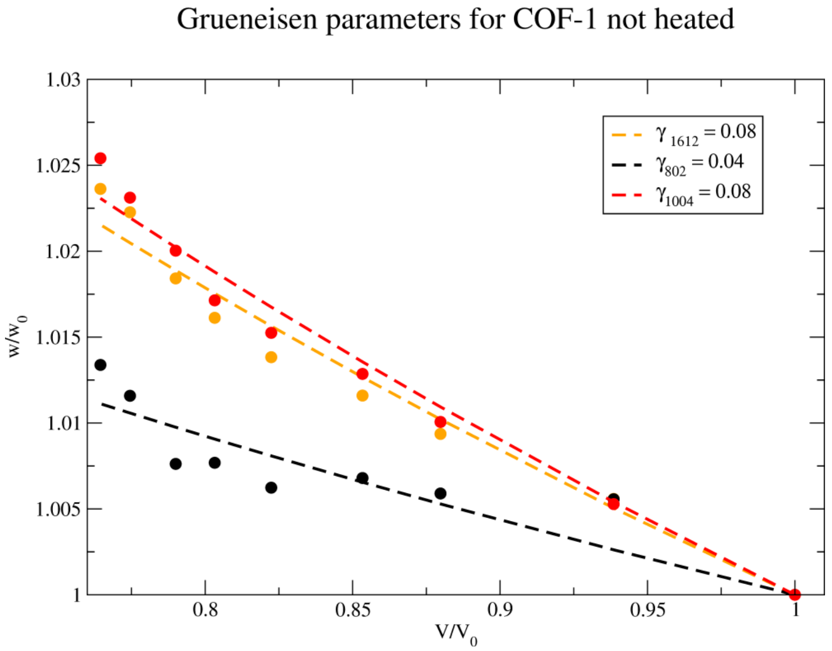

Figure S15. Grueneisen parameters calculated for three strongest COF-1-M Raman modes.

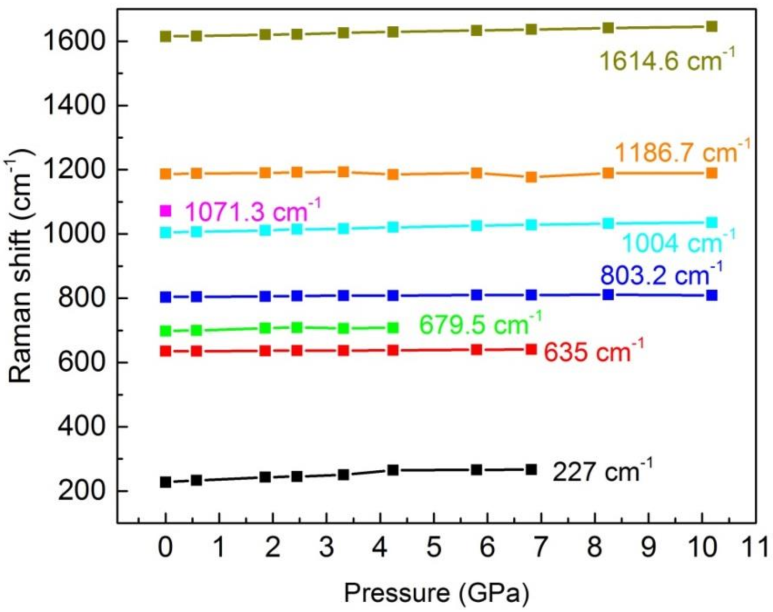

Figure S16. Pressure dependence of Raman peak positions of annealed (empty pore) COF-1. Numbers correspond to peak position observed at highest pressure point when the mode was observed.

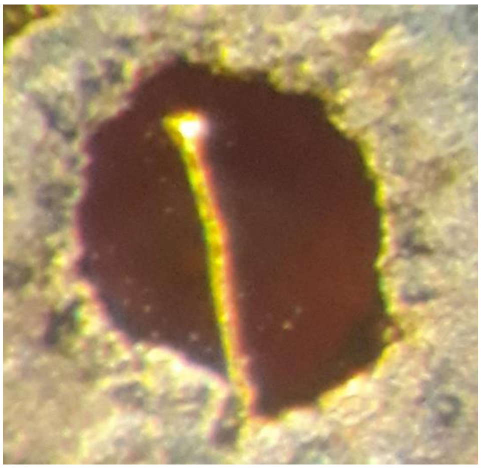

Figure S17 Optical photo of COF-1 inside of DAC taken at 21 GPa. COF-1 is white and transparent material at ambient conditions. It change color to dark brown above ~15 GPa and maintain this color after decompression. Thickness of gold wire is 0.005 mm.

7. FTIR analysis of decompressed samples.

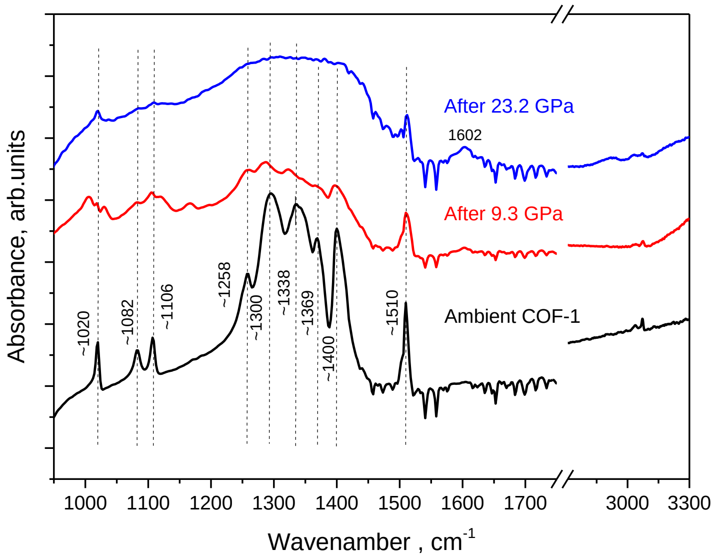

Figure S18. FTIR spectra recorded from precursor COF-1 powder, sample decompressed from 9.3 GPa and sample decompressed from 23.2 GPa using FTIR microscope (Tensor 27) and 15* objective in transmission mode.

The sample decompressed from 9.5 GPa shows all peaks typical for precursor COF-1 thus providing evidence for reversible compression-decompression cycle. Some general broadening of spectra and small shifts by were found for C-C and B-O stretching modes, most likely due to effects of residual stress (see Table 2 for the assignments). Note that pressure 9.3 GPa is just below the pressure point (~10 GPa) where irreversible amorphization of COF-1 begins.

The sample decompressed from 23.2 GPa showed distinctly different FTIR spectra with most of the typical C-C, C-B and C-O peaks merging into one very broad feature. The only well resolved peaks which are found in this spectrum are due to B-C stretch (1022 cm^{-1}) and C=C (1512 cm^{-1}). New relatively broad feature found at 1602 cm^{-1} not observed in the precursor COF-1 and barely visible after decompression from 9.3 GPa is also evident. This peak might be an indication for formation of some graphitic carbon, this suggestion is also compatible with dark brown color of the sample, in contrast to transparent and white COF-1 (and sample decompressed from 9.3 GPa)

The FTIR spectra of sample decompressed from 23.3 GPa provide evidence for irreversible collapse of framework structure. The amorphous phase formed after collapse is likely to be composed by complex mixture of COF-1 fragments. Similar to Raman spectroscopy the FTIR spectra of high pressure amorphous phase showed new spectral features which are compatible with suggestion of formation of graphitic carbon. This suggestion is also supported by the change of sample color from white to dark brown. It should be noted that the spectra shown in Figure S19 were recorded from small pieces of decompressed sample with few µm thickness. The whole sample (~50 µm thickness) was insufficiently transparent to record the spectra. Low transparency of high pressure phase will make problematic *in situ* FTIR study of COF-1 structure collapse above 10 GPa.

Chemical shift, cm^{-1}	Band description
1022	B—C stretch
1080	B—C stretch characteristic boroxine compounds
1105	C—H in plane deformation band for p-substituted benzene
1170	C—H in plane deformation band p-substituted benzene
1260	C—B stretch; could be overlapped with C—C stretch
1300	C—C stretch
1330	B—O stretch
1370	B—O stretch
1400	phenyl ring C=C vibrational mode
1510	Phenyl ring C=C vibrational mode. Strong
3030	Aromatic C—H stretch from phenyl group
3075	Aromatic C—H stretch from phenyl group
3300	O—H stretch from or end –B(OH)2 groups

Table 2. Assignment of FTIR modes of COF-1 according to ref [3]

8. XPS analysis of sample decompressed from 23.2 GPa.

The sample decompressed from 23.2 GPa was taken off the gasket, and studied by XPS using instrument which allows to analyze area witha diameter of ~20 µm. (PHI 5500by Perkin Elmer, Waltham, Massachusetts, USA), with monochromatic Al Ka source (1486.6 eV)). The decompressed samples with few hundreds micrometers were imaged by x-ray excited secondary electron imaging technique which is similar to how a SEM generates a secondary electron image. The use of Scanning X-ray imaging (SXI) image to select analysis areas guarantees that spectroscopic data are collected from the selected feature of interest. The base pressure in the analysis chamber was ca. 10–9 mbar. The samples were mounted on a Gold foil prior to analysis. The acquisition conditions for such high-resolution spectra were 23.5 eV pass energy with the step of 0.1 eV. All spectra were calibrated on C 1s. The recorded photoelectron peaks were curve fitted using the CasaXPS software and assuming a Shirley background.

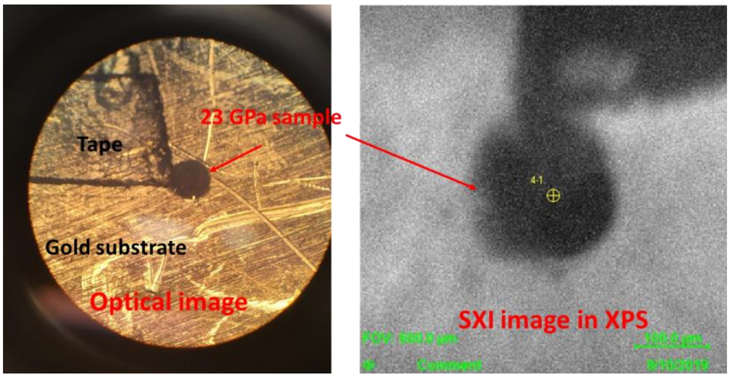

Figure S19 Optical image of decompressed sample mounted for XPS testing and SXS image which shows area used for analysis.

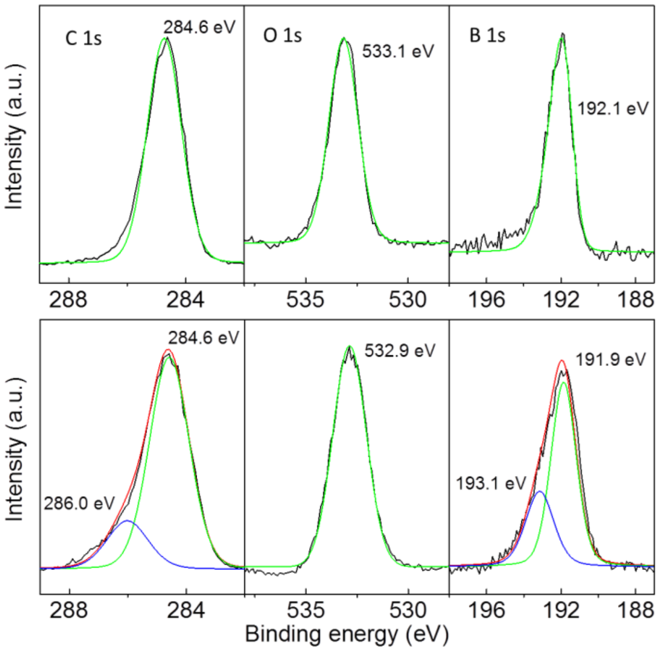

Figure S20 XPS spectra recorded from sample decompressed from 23.2 GPa and reference COF-1 spectrum collected using the same instrument as a reference. The B1s and C1s peaks of decompressed

sample showed distinctly asymmetrical shape with additional (compared to COF-1) components at 193.1 eV and 286.0 eV respectively. The new components indicate at least partial collapse of COF-1 framework structure in amorphous high pressure phase. The O1s peak also become broader and shifted by 0.2 eV.

[1] I. Mosyagin, D. Gambino, D. G. Sangiovanni, I. A. Abrikosov, N. M. Caffrey, *Phys Rev B* **2018**, *98*.

[2] L. Fast, J. M. Wills, B. Johansson, O. Eriksson, *Phys Rev B* **1995**, *51*, 17431-17438.

[3] A. P. Cote, A. I. Benin, N. W. Ockwig, M. O'Keeffe, A. J. Matzger, O. M. Yaghi, *Science* **2005**, *310*, 1166-1170.
